# Spin-orbit-driven magnetic structure and excitation in the 5*d* pyrochlore Cd_2_Os_2_O_7_

**DOI:** 10.1038/ncomms11651

**Published:** 2016-06-07

**Authors:** S. Calder, J. G. Vale, N. A. Bogdanov, X. Liu, C. Donnerer, M. H. Upton, D. Casa, A. H. Said, M. D. Lumsden, Z. Zhao, J. -Q. Yan, D. Mandrus, S. Nishimoto, J. van den Brink, J. P. Hill, D. F. McMorrow, A. D. Christianson

**Affiliations:** 1 Quantum Condensed Matter Division, Oak Ridge National Laboratory, Oak Ridge, Tennessee 37831, USA; 2 London Centre for Nanotechnology, University College London, London WC1H 0AH, UK; 3 Institute for Theoretical Solid State Physics, IFW Dresden, Dresden D01171, Germany; 4 Condensed matter physics and materials science department, Brookhaven National Laboratory, Upton, New York 11973, USA; 5 Beijing National Laboratory for Condensed Matter Physics and Institute of Physics, Chinese Academy of Sciences, Beijing 100190, China; 6 Advanced Photon Source, Argonne National Laboratory, Argonne, Illinois 60439, USA; 7 Department of Materials Science and Engineering, University of Tennessee, Knoxville, Tennessee 37996, USA; 8 Materials Science and Technology Division, Oak Ridge National Laboratory, Oak Ridge, Tennessee 37831, USA; 9 Institute for Theoretical Physics, Technische Universität Dresden, Dresden D01069, Germany; 10 Department of Physics & Astronomy, University of Tennessee, Knoxville, Tennessee 37996, USA

## Abstract

Much consideration has been given to the role of spin-orbit coupling (SOC) in 5*d* oxides, particularly on the formation of novel electronic states and manifested metal-insulator transitions (MITs). SOC plays a dominant role in 5*d*^5^ iridates (Ir^4+^), undergoing MITs both concurrent (pyrochlores) and separated (perovskites) from the onset of magnetic order. However, the role of SOC for other 5*d* configurations is less clear. For example, 5*d*^3^ (Os^5+^) systems are expected to have an orbital singlet with reduced effective SOC. The pyrochlore Cd_2_Os_2_O_7_ nonetheless exhibits a MIT entwined with magnetic order phenomenologically similar to pyrochlore iridates. Here, we resolve the magnetic structure in Cd_2_Os_2_O_7_ with neutron diffraction and then via resonant inelastic X-ray scattering determine the salient electronic and magnetic energy scales controlling the MIT. In particular, SOC plays a subtle role in creating the electronic ground state but drives the magnetic order and emergence of a multiple spin-flip magnetic excitation.

## Abstract (web summary)

Strong electronic correlations in 5*d* materials such as osmates may combine with spin-orbit coupling to yield novel order. Here, the authors demonstrate how spin-orbit coupling in pyrochlore Cd_2_Os_2_O_7_ generates magnetic order and excitations associated with a magnetic metal-insulator transition.

## Graphical abstract

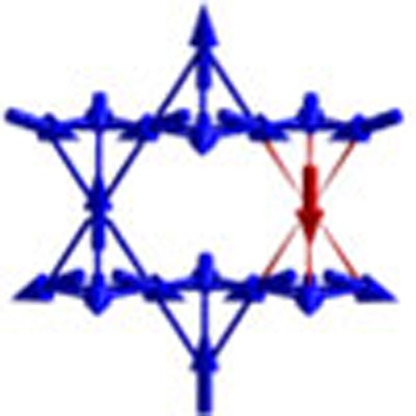

The diverse physics of transition metal oxides has stimulated interest for decades. Particular focus has resided on 3*d* oxides where the strong electron correlations dominate, with a dramatic manifestation being the occurrence of the Mott metal-insulator transition (MIT)^1^,^2^. Contrastingly in 5*d* oxides the relativistic spin-orbit coupling (SOC) is increased to such an extent that competition occurs with the on-site electron correlations, as well as further interactions such as the crystalline electric field (CEF), Hund's coupling and increased orbital overlap. The consequence of these often finely balanced interactions in 5*d* oxides is the emergence of new physics, such as a SOC dominated *j*_eff_=1/2 Mott-like insulating state, initially observed in perovskite iridates^3^,^4^, and Weyl semi-metal, non-trivial topological insulators and magnetic MITs in pyrochlore iridates^2^,^5^,^6^. Considering the pyrochlore osmate Cd_2_Os_2_O_7_, the concomitant magnetic ordering and MIT cannot be reconciled with the Mott–Hubbard paradigm^7^,^8^,^9^,^10^,^11^. Instead the behaviour was initially considered to be the first manifestation of a Slater transition where the onset of magnetic order creates the insulating phase by introducing a periodic potential that localizes the conduction electrons^12^. More recently, however, the mechanism of the MIT has been argued to be a Lifshitz transition normally associated with metal–metal transitions and generally not explicitly requiring magnetic order^7^,^8^.

Phenomenologically the behaviour of Cd_2_Os_2_O_7_ is analogous to iridate pyrochlores: they both undergo temperature dependent continuous MITs suggested to be associated with all-in/all-out (AIAO) magnetic ground states where all spins either point in or out of the centre of the tetrahedron^13^,^14^,^15^. One consequence of the AIAO ground state is that since the ordering breaks time-reversal symmetry, but maintains the cubic symmetry of the lattice, such systems can host Weyl semi-metal behaviour^5^,^6^. Although there exists apparent similarities between osmate and iridate pyrochlores, they contain distinct electronic occupancies of 5*d*^5^ in the iridates and 5*d*^3^ in osmates. One consequence of this is the consideration of SOC in the manifested behaviour of these systems. In iridates SOC assumes an explicit role in the behaviour and is readily evidenced, for example in the creation of the *j*_eff_=1/2, 3/2 electronic ground state. However, in the osmates the influence of similarly large SOC in the creation of the electronic ground state and emergent physical behaviour is often subtle resulting in it often being neglected or considered implicitly.

Here, we consider the role of SOC in Cd_2_Os_2_O_7_ by accessing the electronic and magnetic ground states and the excitations that emerge. In doing so we consider both the similarities and distinctions between 5*d*^3^ and 5*d*^5^-based pyrochlore systems. We access the magnetic Os sublattice in Cd_2_Os_2_O_7_ with neutron powder diffraction (NPD) and confirm the AIAO magnetic structure, thereby going beyond the strong but indirect evidence presented for AIAO ordering of the Ir sublattice in the pyrochlore iridates. To uncover the salient electronic and magnetic interactions, we perform osmium L-edge resonant inelastic X-ray scattering (RIXS) that allows us to directly probe the 5*d* electrons and associated-microscopic interactions, and their energy hierarchy, which we benchmark against detailed theory. These measurements extend the RIXS technique to 5*d* materials beyond iridates, where they have provided important insights^16^,^17^,^18^,^19^,^20^,^21^,^22^. In Cd_2_Os_2_O_7_, we find electronic excitations between 5*d* states that are markedly different from iridates, thereby revealing a divergent and suppressed role of SOC in the creation of the electronic ground state. However, when considering higher order effects or interactions that derive from more than one Os ion, we find an elevated role of SOC in Cd_2_Os_2_O_7_ and similar behaviour to pyrochlore iridates. The subtle but central role of SOC is realized both in the creation of the magnetic ground state and in the observation of a magnetic excitation. The excitation emerges in Cd_2_Os_2_O_7_ due to the cooperation of the large SOC, single-ion anisotropy and Dzyaloshinskii–Moria (DM) interactions that combine to form a superposition of multiple spin-states from the AIAO magnetic ground state. Collectively, despite the distinct electronic ground states, the NPD and RIXS results on the 5*d*^3^-osmate Cd_2_Os_2_O_7_ indicate parallels with 5*d*^5^-iridate pyrochlores and the predictions of associated exotic topological insulating and Weyl semi-metal states.

## Results

### Magnetic ground state of Cd_2_Os_2_O_7_ resolved with NPD

**Figure 1:**
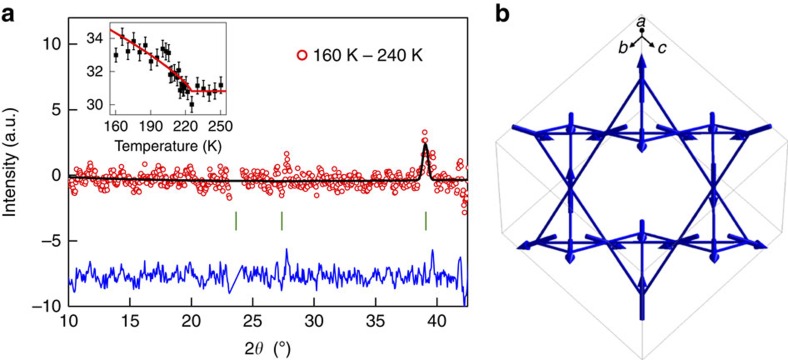
Magnetic structure determination of Cd_2_Os_2_O_7_ with neutron diffraction. (**a**) NPD measurements on ^114^Cd_2_Os_2_O_7_. The ^114^Cd isotope was used to overcome the significant neutron absorption of Cd. The data (red circle) show the difference between measurements taken at 240 and 160 K and was fit with an all-in/all-out magnetic structural model (black line). The blue line is the difference between the measurement and magnetic model. (Inset) The (220) magnetic reflection intensity, 2*θ*=39.1°, shows magnetic ordering occurs at *T*_*N*_=225 K. The error on the data represents the square root of the measured counts. The red line is a guide to the eye derived from a least squares fit to a power law. (**b**) The spins (blue arrows) all point in or out of the centre of the tetrahedron forming the all-in/all-out magnetic structure in Cd_2_Os_2_O_7_. The bonds between Os ions forming the tetrahedra are shown as solid lines.

The specific type of magnetic structure adopted on the frustrated pyrochlore lattice is predicted to play a crucial role in the potential Weyl semi-metal state in the pyrochlore iridates and the magnetic MIT in Cd_2_Os_2_O_7_. The magnetic ground state in Cd_2_Os_2_O_7_ has been shown to be consistent with the AIAO ordering^8^, however, as with the predictions of AIAO ordering on the pyrochlore iridates this relied on some conjecture on the basis of structural arguments. Experimentally both iridium and cadmium-based compounds suffer from strong neutron absorption and coupled with small magnetic moments, this has prohibited the successful observation of magnetic Bragg peaks from the 5*d* ions in either iridate or osmate pyrochlores with NPD. To overcome this, we prepared a Cd_2_Os_2_O_7_ powder sample with isotopic ^114^Cd that can be measured with NPD with negligible neutron absorption. The results of NPD measurements are shown in Fig. 1a. We modelled the data with the allowed irreducible representations (Γ) for a *q*=0 propagation vector and the Os ion at the 16*c* Wyckoff site of (0,0,0) (ref. 8). Only the AIAO magnetic structure model, irreducible representation Γ_3_, accurately fit the data, thereby confirming this as the magnetic ground state formed in Cd_2_Os_2_O_7_. The AIAO magnetic structure is shown in Fig. 1b. By normalizing the magnetic intensity to structural Bragg peaks, we obtained an ordered-magnetic moment of 0.59(8)*μ*_B_/Os. This is significantly reduced from the expected value for a *S*=3/2 system of 3 *μ*_B_/Os. The presence of reduced magnetic moments has been noted in several 5*d* systems, and specifically those with the same 5*d*^3^ occupancy^23^ and is often explained in terms of the extended orbitals in 5*d* systems leading to an increase in hybridization and reduced local moment, and where applicable, frustration. Nevertheless, questions still remain as to whether this is in general a complete description, and specifically if SOC plays a role in the reduction. For example, do cases with a large reduction in the moment in 5*d*^3^ systems indicate an alteration of the electronic ground state away from an orbitally quenched *t*_2g_^3^ state into, for example, an electronic ground state similar to that found in the iridates^3^. In addition, an understanding of the role of SOC, particularly when combined with further effects, such as non-cubic distortions and anisotropies in the system, is required to explain the emergent physical properties out of the AIAO magnetic ground state that appears central to the behaviour in this pyrochlore osmate and related iridate pyrochlores. To gain this understanding, we performed RIXS measurements at the Os L-edge in Cd_2_Os_2_O_7_.

### RIXS measurements of Cd_2_Os_2_O_7_ at the osmium L_3_-edge

**Figure 2:**
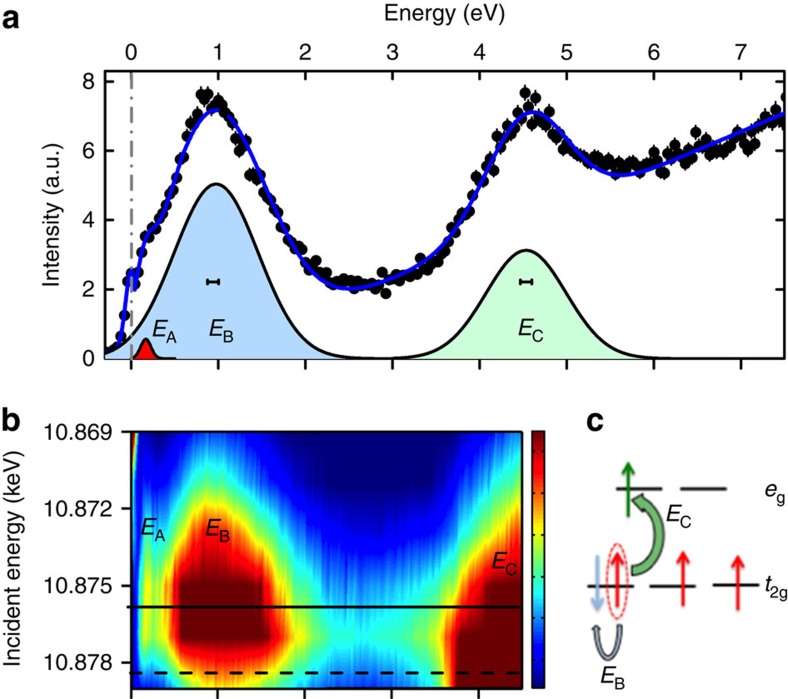
Probing the electronic ground state of Cd_2_Os_2_O_7_ with RIXS at the osmium L-edge. (**a**) Fixed incident energy photons of *E*_*i*_=10.877 KeV, corresponding to the osmium resonant L_3_-edge, probe an inelastic energy loss spectrum out to 7.5 eV (black dots). Three inelastic features are resolvable, *E*_A_=0.16(1) eV, *E*_B_=0.92(6) eV and *E*_C_=4.5(1) eV. The horizontal bars indicate the instrument resolution of 130 meV (FWHM). The full RIXS spectrum was modelled with three Gaussians fitting the inelastic peaks *E*_A_, *E*_B_ and *E*_C_, and a Gaussian for the elastic signal on top of a sloping background (blue line). (**b**) Varying the incident energy to follow the intensity dependence of the inelastic spectra reveals the maximum resonant intensity of *E*_A_ and *E*_B_ occur at the same incident energy of *E*_*i*_=10.8755(5) keV (solid line), whereas *E*_C_ has a maximum at *E*_*i*_=10.879(1) keV (dashed line). The difference of the resonant energies reflects the splitting of the osmium *d*-manifold, nominally into *t*_2g_ and *e*_g_ shells, and allows a categorization of the excitations as intra-*t*_2g_ (*E*_A_ and *E*_B_) or *t*_2g_−*e*_g_ (*E*_C_). (**c**) Schematic of the initial and final RIXS process. The initial electronic ground state before exciting an electron is indicated by the red spins in the limit of cubic CEF splitting of the 5*d* manifold. The final RIXS states for *E*_B_ (intra-*t*_2g_) and *E*_C_ (*t*_2g_−*e*_g_) are indicated by the blue and green spins, respectively. All measurements were performed at 60 K at **q**=(2.5,8.8,8.8).

**Figure 3:**
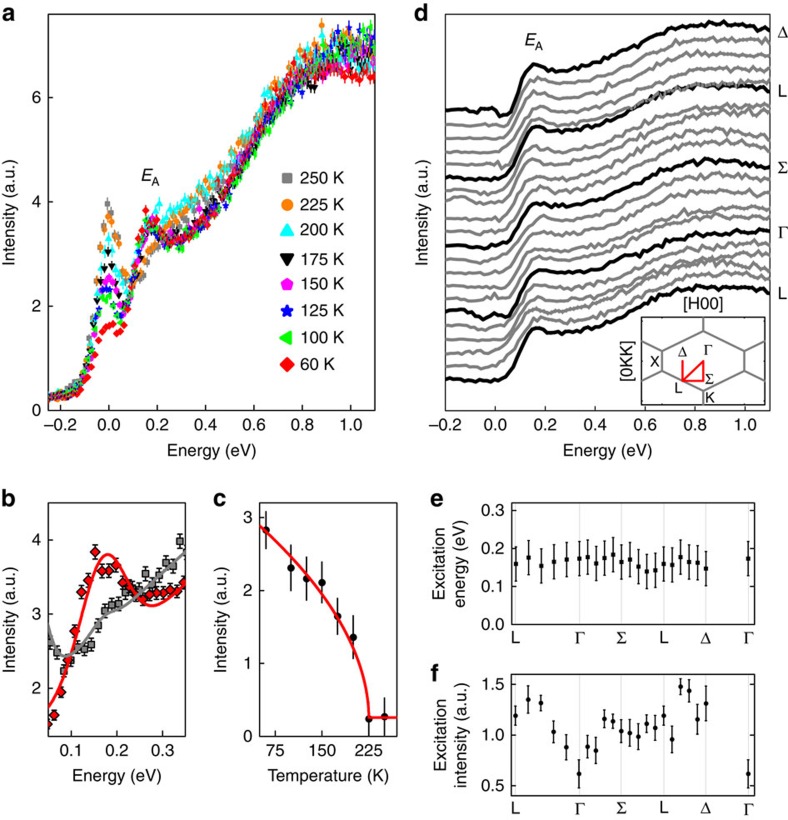
Temperature and momentum dependence of excitation *E*_A_. (**a**) RIXS spectra at constant **q**=(2.5,8.8,8.8) and fixed incident energy *E*_*i*_=10.877 KeV at several temperatures through the magnetic MIT in Cd_2_Os_2_O_7_ reveals *E*_A_ as a temperature dependent excitation. (**b**) The low-energy excitation (*E*_A_=160 meV), along with the elastic line, was fit to a Gaussian on a background from the higher energy scattering at 60 K (red diamonds) and 250 K (grey squares). (**c**) The intensity at *E*_A_ appears and increases below the magnetic MIT temperature of 225 K, the line is a least squares fit to a power law. (**d**) RIXS measurements in the magnetic insulating regime at *T*=60 K performed along high-symmetry directions in the magnetic Brillouin zone. The elastic scattering was suppressed by measuring within 4° of 2*θ*=90°, the remaining elastic signal has been subtracted from the data. (Inset) The Brillouin zone in the (HKK) plane is shown (grey) and the directions measured (red). (**e**) The inelastic energy and (**f**) intensity dependence of *E*_A_ reveal dispersionless behaviour for *E*_A_. Error bars throughout the figure represent the s.d. in the data fitting procedure.

The results for RIXS measurements at fixed incident energy at the Os resonant edge energy of 10.877 KeV are shown in Fig. 2a. Two pronounced features are evident, labelled *E*_B_ and *E*_C_, each significantly broader than the experimental energy resolution and centred at *E*_B_=0.92(6) eV and *E*_C_=4.5(1) eV. In addition, a small, sharp resolution-limited feature *E*_A_ is observed at *E*_A_=0.16(1) eV. (*E*_A_ is more apparent in Fig. 3). Much can be learned by probing the intensity dependence of the inelastic spectra at different fixed incident energies, as shown in Fig. 2b. These measurements reveal features *E*_A_ and *E*_B_ have their maximum resonant intensity at the same incident energy, 10.8755(5) keV, whereas *E*_C_ has a maximum intensity at a higher incident energy of 10.879(1) keV. The resonant energies correspond to the core–hole transition energy accessed during the RIXS process^18^,^19^. For Os L-edge RIXS, the core–hole process is 2*p*–5*d*. However, the 5*d* manifold is nominally split into *t*_2g_ and *e*_g_ sub-manifolds that result in a distinction between the energies of the 2*p*–5*d*(*t*_2g_) and 2*p*–5*d*(*e*_g_) resonant processes. The scattering involving excitations within the *t*_2g_ manifold will occur at a lower energy than scattering involving *e*_g_ levels, with the energy difference corresponding to the *t*_2g_ and *e*_g_ splitting. This allows us to assign features *E*_A_ and *E*_B_ to intra-*t*_2g_ processes and *E*_C_ as involving *t*_2g_–*e*_g_ processes.

### 5*d*-manifold ground state and energy scales in Cd_2_Os_2_O_7_

To further interpret the underlying physical processes and interactions leading to the measured RIXS spectra, we benchmark our results against recent many-body quantum chemistry calculations^24^. Those calculations predicted Os *d*–*d* multiplet excitations in Cd_2_Os_2_O_7_ starting around 1.5 eV, which overestimates the energy but is generally consistent with the measured energy for *E*_B_ (we discuss this divergence more when addressing excitation *E*_C_). Moreover, this excitation is predicted to be from intra-*t*_2g_ processes, agreeing with the results in Fig. 2b. In the absence of SOC and trigonal CEF distortion, *E*_B_ will consist of three peaks, however, these are unresolvable. Including SOC and trigonal CEF will split these peaks further^24^. However, the single-broad feature of *E*_B_ indicates this splitting is not appreciable. As shown schematically in Fig. 2c, this intra-*t*_2g_ (*t*_2g_^3^→*t*_2g_^3^) excitation at *E*_B_ can be described as corresponding to a spin flip of one of the three electrons in the 5*d*^3^ valance band at the osmium site, changing the total local spin from high-spin *S*=3/2 to low-spin *S*=1/2, and reveals information on the *t*_2*g*_ splitting and Hund's coupling (*J*_H_). Even considering the atomic level, these states have multi-determinant character and requires careful interpretation^25^. For example, to form an eigenstate the determinant shown in the Fig. 2c (↓↑↑) needs to be augmented by the (↑↓↑) determinant with a minus sign. The corresponding energy of this multi-determinant state is 3*U*′, while the single low-spin determinant has the energy 3*U*′-*J*_H_ and the high-spin single-determinant state has 3*U*′-3*J*_H_ (following the notation of ref. 25). Overall, peak *E*_B_ hosts in total eight *S*=1/2 states (neglecting SOC and trigonal distortions to first approximation)^24^. Five of those have relative energy of 3*J*_H_ and the remaining three have 5*J*_H_ causing the average to be 3.75*J*_H_. This gives an estimate of *J*_H_=0.92 eV/3.75≈0.25 eV.

This type of Hund (spin) excitation, which has been observed in 3*d* oxides, has never been previously measured in a 5*d* system. Indeed the presence of *E*_B_ immediately reveals a different *d*-manifold electronic ground state in Cd_2_Os_2_O_7_ compared to the *j*_eff_=1/2 Mott-like iridates. Specifically, Ir L-edge RIXS measurements show appreciable SOC splitting of the *t*_2g_ manifold, of the order 0.4 eV in pyrochlore iridates^22^ in the form of a spin-orbit exciton that is absent from the RIXS spectra for Cd_2_Os_2_O_7_ (refs 16, 17, 18, 19, 20). The absence of a SOC-driven exciton in the RIXS spectra of Cd_2_Os_2_O_7_, with instead the presence of *E*_B_, strongly indicates that SOC does not appreciably split the *t*_2g_ manifold, that is, the SOC-driven *j*_eff_=3/2, 1/2 electronic ground state is not realized in this 5*d*^3^ osmate.

The higher energy excitation *E*_C_ involves transitions that access the *e*_g_ level, as shown in Fig. 2b. Calculations predicted inter *t*_2g_-*e*_g_ excitations (*t*_2g_^3^→*t*_2g_^2^*e*_g_^1^) at 5 eV (ref. 24), again about 0.5 eV above the observed RIXS peak indicating a consistent overestimation in the calculations. The divergence between predicted and observed excitations, at least in part, can be explained by the details of the calculations in ref. 24, where altering the number of states or description of the nearest neighbour sites in the spin-orbit treatment will affect the predicted energies. Excitation *E*_C_, shown schematically in Fig. 2c, is a direct measurement of the 10*Dq* CEF splitting of 4.5 eV in Cd_2_Os_2_O_7_. For SOC to play a role in the ground state electronic configuration it needs to intermix the *t*_2g_^3^ manifold itself, which our measurement of feature *E*_B_ indicates does not appreciably occur, or mix it with *t*_2g_^2^*e*_g_^1^ states. However, with a large 10*Dq* splitting of 4.5 eV, of order 1 eV larger than that found in pyrochlore iridates^22^, the latter mixing is strongly prohibited.

Collectively the RIXS results for *d*–*d* transitions in Cd_2_Os_2_O_7_ reveal an electronic ground state of the 5*d*^3^ ion in Cd_2_Os_2_O_7_ that is dominated by CEF (4.5 eV) followed by Hund's coupling (0.3 eV). The much reduced effect of SOC in the creation of the electronic ground state of the Os^5+^ ion, counter intuitively, is not at odds with the expectations of observable effects of the large SOC that is intrinsic to 5*d* systems^7^,^11^. For example, as we discuss further, the role of SOC comes to the fore when either considering excited magnetic and electronic states or when going beyond the single-ion ground state.

### Momentum and temperature dependence of excitation *E*_A_

Having characterized the electronic ground state from RIXS excitations at 1 eV and above, we now focus on feature *E*_A_ at 160 meV. This energy does not correspond to any expected *d*–*d* energy scale for the *d*^3^ electronic configuration in a nearly cubic CEF, see for example, ref. 26. Moreover, *E*_A_ is distinct from the *d*–*d* excitations *E*_B_ and *E*_C_ in being much sharper in energy. Figure 3a–c shows the intensity of *E*_A_ follows an order parameter-like behaviour with temperature, with *E*_A_ appearing at the magnetic MIT, and remains at the same energy of 160 meV. We followed the momentum dependence, Fig. 3d,e, and conclude *E*_A_ is a non-dispersive excitation, within the 130 meV experimental resolution. While the intensity of *E*_A_ appears to show some variation within the Brillouin zone (Fig. 3f), this is, at least in part, an artifact of the observed variation of the elastic line as the crystal is necessarily measured in slightly different physical orientations altering the X-ray beam attenuation.

The origin and behaviour of mode *E*_A_ is puzzling for a variety of reasons. Interpretation of this feature in terms of a conventional magnetic excitation appears problematic given the small calculated value of the nearest-neighbour exchange interaction *J*=6.4 meV for Cd_2_Os_2_O_7_ (ref. 24). Therefore, we first consider potential non-magnetic mechanisms. One scenario, given the concomitant MIT, is a relationship with the insulating state. Such an excitation was observed in the iridate A_2_IrO_3_ (ref. 20), with an excitation of 340 meV corresponding exactly to the Mott-gap size. A similar origin, however, is inconsistent with known behaviour of Cd_2_Os_2_O_7_, for which the insulating gap has been shown to open continuously, a fact that has been cited in favour of a Slater mechanism^7^,^8^. Therefore, if feature *E*_A_ was related to the insulating gap, one would expect to observe a significant shift in the energy with temperature. This is not the case. Moreover, the insulating gap, 2Δ, in Cd_2_Os_2_O_7_ is around 100 meV (ref. 8) and is, therefore, not consistent with the measured RIXS spectra. We finally rule out a *d*–*d* excitation scenario by noting that the *t*_2g_ levels are already split at high temperature (>225 K) in Cd_2_Os_2_O_7_, indeed this is one of the causes of the single-ion anisotropy in the system. Any alteration of the *t*_2g_ splitting due to lower symmetry crystal fields below the magnetic MIT will not produce such a low-lying *d*–*d* excitation. This is supported by the RIXS measurements not showing any change in *E*_B_ or *E*_C_ that would indicate an altered ground state and potential routes for *E*_A_.

### SOC-driven magnetic excitation

We argue that *E*_A_ does indeed have a magnetic origin and support this with numerical exact diagonalisation (ED) calculations. The AIAO magnetic structure is stabilized in the frustrated pyrochlore lattice by single-ion axial anisotropy^7^,^8^,^24^. Therefore, in the leading approximation the spins can be considered to be Ising-like local *S*=3/2. In this case, the resultant classical Hamiltonian that includes easy-axis anisotropy (*D*), Heisenberg exchange (*J*) and the DM interaction (*d*) gives three types of local-spin excitations for this *S*=3/2 system of *S*_*z*_=3/2→1/2 (Δ*S*_*z*_=1), *S*_*z*_=3/2→−1/2 (Δ*S*_*z*_=2) and *S*_*z*_=3/2→−3/2 (Δ*S*_*z*_=3). These have the following discrete energies:


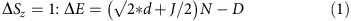



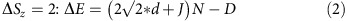



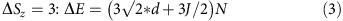


**Figure 4:**
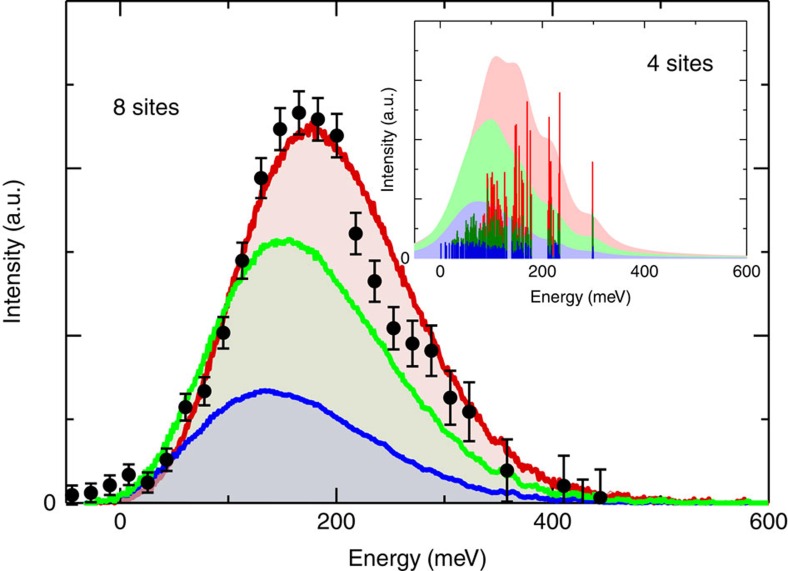
Spin-orbit-driven magnetic excitation from an AIAO ground state in Cd_2_Os_2_O_7_. Eight site ED calculations, including resolution broadening, showing the character and amount of excited eigenstates at a particular energy. The scaled-experimental data (black) is shown after background subtraction with error bars representing 1 s.d. The ED results reveal Δ*S*_*z*_=1,2,3 (*Ô*=*S*_+_,(*S*_+_)^2^,(*S*_+_)^3^) excitations indicated by blue, green and red, respectively. The strong SOC and consequently DM interactions in the magnetic ground state and excited states of Cd_2_Os_2_O_7_ result in a superposition of these Δ*S*_*z*_ spin-states all contributing to the excitation. The calculated intensity corresponds to the DOS and therefore the actual experimental intensity distribution of the excitation measured by the RIXS cross section is likely dominated by the Δ*S*_*z*_=1 process, followed by Δ*S*_*z*_=2 and then Δ*S*_*z*_=3. Inset corresponds to 4-site calculations and highlights the mixing and energy distribution of the Δ*S*_*z*_ excitations.

To explore the relevance of the Δ*S*_*z*_ processes to the RIXS data, we performed ED calculations. The ground state and all possible excited states of the Hamiltonian


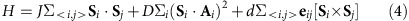


with unitary vectors 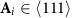 and 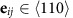 were obtained for given 4-site and 8-site clusters that encompass the full AIAO magnetic ground state with a fixed parameter set reported in ref. 24, that includes *d*=1.7 meV, *D*=−6.8 meV and *J*=6.4 meV. The ED calculations take into account explicitly the quantum nature of the spin-states and the interactions between them, the results are shown in Fig. 4. The calculated energy shows excellent agreement with experiment. We note that the intensities calculated are the density of states (DOS) and therefore are not directly proportional to the RIXS cross section, which is non-trivial to calculate.

In the classical limit, equations (1, 2, 3), the possible spin-flip excitations for a *S*=3/2 system of Δ*S*_*z*_=1, 2, 3 have distinct energies. Conversely, the spectra for the Δ*S*_*z*_ excitations from ED calculations are mixed and rather similar with an overlapping single-peaked excitation. The only distinction between the different Δ*S*_*z*_ processes from ED calculations is a slight variation in energy and an overall intensity-scaling factor in their DOS, as shown by the red, green and blue regions in Fig. 4. The mixing of Δ*S*_*z*_=1, 2, 3 is due to quantum fluctuations leading to a superposition of different spin-states that occurs due to the DM interaction being appreciable in strength in Cd_2_Os_2_O_7_, *J*/*d*=3, which is in turn related to the strong SOC intrinsic in Cd_2_Os_2_O_7_. The DM interaction mixes states with different spin projection *S*_z_ into both the ground state and the excited states. This mixing occurs to such an extent that the separate Δ*S*_*z*_=1, 2, 3 excitation channels become indistinguishable and result in spectra with broad tails and a maximum of intensity very close to the energy measured for *E*_A_ of 160 meV.

The strong agreement between the observed magnetic excitation energy and that predicted from ED calculations on the basis of the inclusion of the *J*, *d* and *D* interactions predicted in ref. 24, with essentially no free parameters, indicates that this model robustly describes the essential physics of the system. Further experimental support for the magnetic origin of excitation *E*_A_ is found when recalling the RIXS incident-energy dependence (Fig. 2b). The incident energy spectra showed both *E*_A_ and *E*_B_ had the same incident-energy resonance dependence indicating they both involve solely intra-*t*_2g_ excitations. In addition, we observed *E*_A_ is non-dispersive (Fig. 3d). This is indeed expected in a system that is predominantly of Ising type, since the AIAO structure is a lowest energy, rather than degenerate, ground state, and will suppress the propagation of a flipped spin that alters the magnetic ordering.

Inspecting the ED calculations in Fig. 4 shows the prediction of the strongest DOS contribution to be from the Δ*S*_z_=3 process. Such an excitation would usually be forbidden by RIXS spin-only selection rules that limits the possible measurable excitations to Δ*S*_*z*_=1 and 2 (ref. 27). The Δ*S*_*z*_=3 process, however, becomes experimentally allowed in Cd_2_Os_2_O_7_ in the intermediate RIXS process due to the creation of a 5*d*^4^ state on the Os ion (2*p*^6^5*d*^3^→2*p*^5^5*d*^4^) where SOC will dominate. In this intermediate state *S* is no longer a good quantum number, therefore the standard spin-only selection rules no longer apply making a Δ*S*_*z*_=3 excitation allowed and measurable with RIXS. Indeed this would be the first such case of a measured excitation with a Δ*S*_*z*_=3 component by any experimental technique, for example neutrons carry spin-1/2 and can only measure Δ*S*_*z*_=0 or 1 magnetic excitations. Nevertheless, the experimental RIXS cross section will expected to be dominated by the single-magnon followed by the two-magnon processes and then the Δ*S*_*z*_=3 triple-magnon component.

## Discussion

The NPD and Os L-edge RIXS measurements have provided direct access to the 5*d* electrons, competing inter and intra-ion interactions, and probed the AIAO magnetic ground state and subsequent excitations in Cd_2_Os_2_O_7_. The results allow a detailed and general understanding of the role of SOC and magnetism in creating the MITs in both osmate (5*d*^3^) and iridate (5*d*^5^) pyrochlores where the behaviour is phenomenologically similar, but the electronic ground state is revealed as distinctly different.

First, we consider the electronic ground state. In the 5*d*^5^ iridates, there is one hole in the *t*_2g_ shell giving rise to three nearly degenerate orbital configurations carrying orbital moment *l*_eff_=1 and spin *S*=1/2, which SOC splits into a *j*_eff_=1/2 doublet and a *j*_eff_=3/2 quartet. This splitting has been observed in several RIXS measurements as a well-defined feature termed a spin-orbit exciton^16^,^17^,^22^. Conversely in Cd_2_Os_2_O_7_, there are three valence electrons that half-fill the *t*_2g_ shell. Therefore, to the leading order Cd_2_Os_2_O_7_ carries a spin *S*=3/2 and no orbital moment *l*_eff_=0. The orbital moment is quenched because there is only one possible orbital configuration with three spins aligned. The RIXS spectra of Cd_2_Os_2_O_7_ provides direct evidence of the different ground states in 5*d*^5^ (Ir) compared to 5*d*^3^ (Os) via the measurement of excitation *E*_B_ in Cd_2_Os_2_O_7_ that reveals a *t*_2g_ manifold with no observable splitting into *j*_eff_=3/2,1/2 bands. This occurs since SOC cannot affect the half-filled *t*_2g_ shell, apart from second-order effects. In this strict sense, SOC plays a negligible role in creating the electronic *S*=3/2 ground state in 5*d*^3^ systems. Considering second-order effects in Cd_2_Os_2_O_7_, the leading correction comes from the fact that the *e*_g_ states, while well-separated, are not infinitely far above the *t*_2g_ shell. SOC can therefore admix *e*_g_ states into the *t*_2g_ *S*=3/2 ground state. This effect is governed by the ratio of the SOC *λ* (∼0.4 eV) (ref. 6) and the crystal field splitting 10*Dq* (∼4.5 eV). The leading order result of this mixing is a single-ion magnetic anisotropy: a splitting between the *S*_*z*_=+/−1/2 and the *S*_*z*_=+/−3/2 projections of the *S*=3/2 manifold, where *z* is the easy axis of the system. For Cd_2_Os_2_O_7_ this results in an easy-axis anisotropy of around 7 meV (ref. 24). Further consequences of the SOC exist when going beyond single-site considerations, such as the DM interaction. Therefore, while SOC does not dominate the mechanisms creating the electronic ground state of a single Os ion, in contrast to the *j*_eff_=1/2 Mott-like iridates, it can still strongly impact the physics of Cd_2_Os_2_O_7_.

The intrinsically large SOC in Cd_2_Os_2_O_7_ manifests in the stabilization of the AIAO magnetic ground state that was confirmed with NPD measurements. This non-degenerate magnetic ground state is selected in the frustrated pyrochlore structure due to the single-ion anisotropy in the system. A similar mechanism is expected to exist in pyrochlore iridates, although the AIAO ordering has not been directly measured on the Ir sublattice. Nevertheless, the AIAO magnetic ordering has been the focus of considerable interest in pyrochlore iridates due to the potential for exotic phenomena including Weyl semi-metal and topological insulating behaviour^2^,^6^,^13^,^14^,^15^. Analogous behaviour can be mapped over to pyrochlore osmates with AIAO ordering. In terms of the observed MITs in pyrochlore iridates, the AIAO ordering is predicted to play a direct role with the existence of either concurrent or proximate magnetic ordering^6^. Considering the phenomenologically similar behaviour between the pyrochlore iridates and the pyrochlore osmate Cd_2_Os_2_O_7_ suggests an analogous underlying mechanism for the magnetic MITs. Our results indicate neither of the divergent electronic ground states adopted within the 5*d*^3^ osmate and 5*d*^5^ iridate pyrochlore systems of orbital singlet with nearly quenched effective SOC and SOC-enhanced *j*_eff_=1/2, respectively, play a dominant role with regards to creating the MITs. Instead the underlying mechanism appears directly related to the enhanced SOC in both systems that produces the strong single-ion anisotropy and subsequent AIAO magnetic ordering.

The emergence of the magnetic excitation at *E*_A_=160 meV in the RIXS measurements of Cd_2_Os_2_O_7_ provides a measurable manifestation for the large SOC in 5*d*^3^ systems. The existence of this excitation from the AIAO ground state was shown to be a direct consequence of the strong DM exchange interaction and single-ion axial anisotropy that couple with the comparable energy of the magnetic exchange interaction; with the concomitant behaviour necessary to create the observed magnetic excitation. The coupling of these interactions on the Ising-like AIAO ground state results in a magnetic excitation that corresponds to a superposition of multiple spin-flip processes (Δ*S*_*z*_=1, 2, 3) that obeys the Hamiltonian described in equation (4), which includes comparable DM, single-ion axial anisotropy and magnetic exchange interactions.

Collectively, the neutron diffraction and RIXS measurements on Cd_2_Os_2_O_7_ have provided a unique and direct probe of the 5*d* electrons responsible for the concurrent magnetic order and MIT in both the metallic and insulating regimes, and accessed the role of SOC. The results revealed a mechanism of strong CEF (10*Dq*≈4.5 eV), moderate Hunds coupling (*J*_H_≈0.3 eV) and reduced effective SOC in the creation of the electronic ground state. While the SOC does not appreciably influence the electronic ground state manifold, it enhances the anisotropic magnetic couplings to create an AIAO magnetic ground state and the magnetic excitation out of this ground state at the MIT that corresponds to a superposition of multiple spin-flip processes. This reveals a non-trivial and variable role of effective SOC in 5*d*^3^-based systems compared to 5*d*^5^ systems, such as the Ir^4+^ iridates, where SOC plays a more consistently dominant role. The behaviour in Cd_2_Os_2_O_7_ suggests that in general a route beyond the Mott–Hubbard paradigm is required to open the insulating gap in 5*d* pyrochlores, with AIAO magnetism the likely driving mechanism.

## Methods

### NPD measurements

NPD measurements were performed on the HB-2A powder diffractometer at the High Flux Isotope Reactor (HFIR), Oak Ridge National Laboratory^28^. Isotopic ^114^Cd_2_Os_2_O_7_ was used to overcome the substantial neutron absorption from elemental cadmium. Measurements were performed using a wavelength of 2.41 Å between 4 and 250 K. The magnetic structure was refined using Fullprof and SARAh^29^ utilizing an irreducible representational analysis approach. The Γ_3_ irreducible representation was used for the final solution, after trying all possible irreducible representation solutions for a propagation vector of *k*=(0,0,0), and finding this to be the only solution that described the data. The solution corresponds to the all-in/all-out magnetic structure for the magnetic Os ion at the (0,0,0) site in the pyrochlore structure. This corresponds to the magnetic space group Fd-3m' in Belov-Neronova-Smirnova (BNS) notation and standard setting. We obtained the ordered-magnetic moment from the Fullprof refinement by normalizing the magnetic scattering to the nuclear reflections and further checked the lower and upper error bounds were an accurate reflection of the data.

### Resonant inelastic X-ray scattering

Single crystals of Cd_2_Os_2_O_7_ were measured with RIXS at the Os L_3_-edge (10.88 KeV) on Sector 30 at the Advanced Photon Source (APS) using the MERIX instrumentation^30^. The incident energy was accessed with two pre-sample monochromators, a primary diamond(111) monochromator and a secondary Si(400) monochromator. The energy of the beam scattered from the sample was discriminated with a Si(466) 2m diced analyzer. The detector was a MYTHEN strip detector. To reduce the elastic line, we performed inelastic measurements in horizontal geometry within a few degrees of 90°. The RIXS energy resolution was 130 meV FWHM. This is comparable to initial RIXS measurements on iridates that resolved both the SOC splitting of the *t*_2*g*_ bands into *j*_eff_=1/2 and *j*_eff_=3/2 shells, and dispersive magnetic excitations^16^. The Cd_2_Os_2_O_7_ single crystal was oriented in the (HKK) scattering plane to access high-symmetry directions in the Brillouin zone. Measurements were performed in the same Brillouin zone, and all measurements were performed more than once. The fitting described in the text was on the basis of a least squares analysis. For the dispersion relation in Fig. 3e nominal error of 50 meV has been reported, that is larger than the error obtained by least squares, to account for the variation of the elastic line.

### Full exact diagonalisation calculations

The ground state and all possible excited states of the Hamiltonian (equation (4)) with unitary vectors 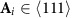 and 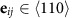 were obtained for given 8-site and 4-site clusters with a parameter set reported in ref. 24. Using the states we calculated the excitation spectrum *I*(*ω*)=*S*_*n*_|<*y*_*n*_|*Ô*| *y*_0_>|^2^d(*ω*−*E*_*n*_+*E*_0_), where E_*n*_ are the *n*-th eigenstate and eigenenergy (*n*=0 corresponds to the ground state). The applied operator was taken as *Ô*=*S*_*+*_, (*S*_*+*_)^2^, (*S*_*+*_)^3^ for Δ*S*_*z*_=1, 2, 3 excitation channels, respectively. Additional calculations were run on a 4-site cluster, which is the minimum to reproduce the all-in/all-out magnetic structure, to test for finite size effects. The results between the 4-site and 8-site showed very little difference.

### Synthesis

Single crystals of Cd_2_Os_2_O_7_ of approximate dimensions 0.2 mm^3^ were grown as described in ref. 9 by sealing appropriate quantities of CdO, Os and KClO_3_ in a silica tube and heating at 800 °C for 1  week. Before the RIXS measurement, several crystals were characterized and aligned with an X-ray Laue. All RIXS measurements were performed on a single crystal of Cd_2_Os_2_O_7_. Polycrystalline ^114^Cd_2_Os_2_O_7_ was prepared with isotopic ^114^Cd for neutron measurements to negate the extremely high-neutron absorption of standard Cd using solid state techniques from ^114^CdO and OsO_2_ powders.

### Data availability

The data that support the findings of this study are available from the corresponding author upon request.

## Additional information

**How to cite this article:** Calder, S. *et al*. Spin-orbit-driven magnetic structure and excitation in the 5*d* pyrochlore Cd_2_Os_2_O_7_. *Nat. Commun.* 7:11651 doi: 10.1038/ncomms11651 (2016).
